# SNPPhenA: a corpus for extracting ranked associations of single-nucleotide polymorphisms and phenotypes from literature

**DOI:** 10.1186/s13326-017-0116-2

**Published:** 2017-04-07

**Authors:** Behrouz Bokharaeian, Alberto Diaz, Nasrin Taghizadeh, Hamidreza Chitsaz, Ramyar Chavoshinejad

**Affiliations:** 1 Facultad informatica, Complutense University of Madrid, Calle Profesor José García Santesmases, 9, 28040 Madrid, Spain; 2 School of Electrical and Computer Engineering, College of Engineering, University of Tehran, Tehran, Iran; 3 Department of Computer Science, Colorado State University, Fort Collins, CO 80523 USA; 4 External Collaborator, Reproductive Biomedicine Research Center, Royan Institute for Reproductive Biomedicine, Tehran, Iran

**Keywords:** SNP, Phenotype, Relation extraction, Negation, Modality, Degree of confidence

## Abstract

**Background:**

Single Nucleotide Polymorphisms (SNPs) are among the most important types of genetic variations influencing common diseases and phenotypes. Recently, some corpora and methods have been developed with the purpose of extracting mutations and diseases from texts. However, there is no available corpus, for extracting associations from texts, that is annotated with linguistic-based negation, modality markers, neutral candidates, and confidence level of associations.

**Method:**

In this research, different steps were presented so as to produce the SNPPhenA corpus. They include automatic Named Entity Recognition (NER) followed by the manual annotation of SNP and phenotype names, annotation of the SNP-phenotype associations and their level of confidence, as well as modality markers. Moreover, the produced corpus was annotated with negation scopes and cues as well as neutral candidates that play crucial role as far as negation and the modality phenomenon in relation to extraction tasks.

**Result:**

The agreement between annotators was measured by Cohen’s Kappa coefficient where the resulting scores indicated the reliability of the corpus. The Kappa score was 0.79 for annotating the associations and 0.80 for the confidence degree of associations. Further presented were the basic statistics of the annotated features of the corpus in addition to the results of our first experiments related to the extraction of ranked SNP-Phenotype associations. The prepared guideline documents render the corpus more convenient and facile to use. The corpus, guidelines and inter-annotator agreement analysis are available on the website of the corpus: http://nil.fdi.ucm.es/?q=node/639.

**Conclusion:**

Specifying the confidence degree of SNP-phenotype associations from articles helps identify the strength of associations that could in turn assist genomics scientists in determining phenotypic plasticity and the importance of environmental factors. What is more, our first experiments with the corpus show that linguistic-based confidence alongside other non-linguistic features can be utilized in order to estimate the strength of the observed SNP-phenotype associations. Trial Registration: Not Applicable

**Electronic supplementary material:**

The online version of this article (doi:10.1186/s13326-017-0116-2) contains supplementary material, which is available to authorized users.

## Background

### Background

An SNP is a single base mutation occurring at the DNA level. Variations in DNA sequences can affect how humans develop diseases and respond to pathogens, chemicals, drugs, and other agents [1]. There exist an approximate ten to thirty million SNPs in humans [2]. As a result of the increasing number of related articles, the use of automatic association extraction in determining the associations of mutations (e.g. SNP’s) and their consequences is increasing in biological systems and genotype-phenotype studies.

In genetic epidemiology, GWA study refers to the process of examining several common genetic variants in different people so as to discover a possible correlation between a variant and a phenotype trait. A phenotype is an organism’s recognizable characteristics or traits such as its development, biochemical or physiological properties, behavior, and the concomitant products of that behavior [3]. The large amount of data generated from these studies [4] necessitates the need to develop an automatic approach in order to facilitate the study of the extracted associations. Recently, a few corpora and methods have been developed with the aim of extracting mutation and disease associations from texts such as [5] and [6]. There is, on the other hand, no available corpus for extracting the association of SNP-phenotypes from texts annotated with negation, modality, and the confidence degree of such associations. The need for different levels of annotation for biomedical associations has been considered in certain biomedical resources such as PharmGKB [7]. It collects information about the impact of human genetic variations in drug responses that have been annotated with four levels of evidence.

In this paper, we described and discussed the process of constructing ranked SNP-phenotype association corpus (SNPPhenA), inter-annotator agreement analyses and the results of some utilized baseline methods during an initial experiment. In most cases, implementing a biomedical text-mining system is a difficult task as the basic scientific communication components — i.e. journals and databases — are designed to be read by humans, not machines or computers. In order to address this problem, xml was selected as the main format for the produced corpus. Furthermore, biomedical Natural Language Processing (BioNLP) systems (e.g. relation extraction) have been mostly applied to abstracts as, though concise, they are more readily available. Also, abstracts are deemed as good targets for information extraction (IE) because they are a succinct and summarized version of an article [8], hence the selection of abstracts in the present research.

### Motivation

Several named entities have been investigated during the biomedical relation extraction task, few of which are suitable candidates for annotating with confidence degrees, which is the major aim of the research when identifying the strength (severity) of associations or interactions. The reason for this is that there are no adequate biomedical agreements. For instance, Drug-drug Interactions (DDI) or Protein-protein Interactions (PPI) are two biomedical relations discussed by a myriad of researchers. However, it is difficult even for a human expert to reliably classify the strength or severity of DDIs or PPIs according to confidence level, a problem existing due to the variation in the types of related experiments and the paucity associated with the methods of quantifying and estimating the significance of both the research method and the association. Most GWA studies that report SNP-phenotype associations are generally based on case-control researches [9] initially tested for statistically significant differences between the proportion of exposed subjects among cases and controls. Accordingly, to gauge the research significance of the result, researchers are encouraged to, more often than not, report a level of evidence by considering *p*-values and study size.

Both preparing a reliable corpus annotated with confidence level in associations and developing an automated tool for this purpose are evidently more difficult for a host of other biomedical named entities that may require different models of study [7]. For instance, comparing and finding an acceptable agreement of confidence level for an association reported in a case-control experiment beside to a case study reported association would be more difficult and challenging. In addition, it is difficult to identify the strength and severity of associations (or interactions) in a sentence explaining a biochemical mechanism occurring in many corpora such as DDI and Protein-related associations because every chemical reaction may precipitate different sequences within the body.

Consequently, insofar as NLP, ranked SNP-phenotype association extraction based on confidence level is considered to be a more feasible task in comparison with many other biomedical association extraction tasks. Additionally, it is worth mentioning that specifying neutral candidates and the effects of negation annotated in the corpus is influenced by measured confidence level of association between two entities, elaborated in the following sections. This shows how crucial it is to have reliable annotations for confidence level in associations as well as an automated method for identifying them.

Yet another objective of the present was to identify the association of such phenotypes as quantitative traits instead of diseases with SNP’s, variously studied by researchers. Such extension is significant because many phenotypes can be detected during the sub-clinical phase of a disease history, hence determining their association with an SNP entails a more early diagnosis and treatment of the disease. Certain phenotypes, it should be noted, are important risk factors for the disease.

### Related tasks and phenomena

**Fig. 1:**
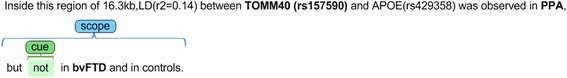
A sample sentence in the corpus within a negation cue and scope

One of the linguistic-based phenomena discussed in this paper is **negation**. According to linguistics [10], negation refers to a morphosyntactic operation wherein a lexical item or construction is denied or whose meaning becomes inverted by another lexical item. Likewise, the lexical item representing the negation is referred to as the negator. Commonly used in clinical and biomedical text documents, negation is a significant cause of low precision in automated information retrieval systems. In the prepared corpus, the marked sentences were annotated with negation scopes and cues. A sample of a negated sentence can be found in Fig. 1, wherein the SNP and phenotypes are written in bold font.

**Fig. 2:**
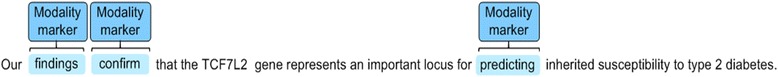
A sample of a sentence with three modality markers

The other linguistically-driven phenomenon employed here is linguistic **modality**. Generally, modal expressions are words that state modality which is the expression of the subjective attitudes and opinions of the presenter about a possible fact or to control a probable action including intentions, possibility, probability, necessity, obligation [11]. In this research, linguistic-based modals and speculation analyses were made use of in order to determine the confidence level of the SNP-phenotype association candidates in the corpus. The linguistic-based confidence level of an extracted biomedical association can provide an estimate for the reliability of the obtained association and the strength of the biomedical association. Figure 2 demonstrates the sample of a sentence in the corpus with three modality markers. The modality analysis of a sentence and the linguistic-based confidence level of associations can be utilized in addition to other non-linguistic features so as to obtain more accurate annotations.

Named Entity Recognition (**NER**) is the first step towards extracting associations and relations as well as making related corpora within biomedical texts [12]. It is crucial to notice that the characteristics of NER in the biomedical domain are different from those in the newswire domain [13]. Identifying mutations in texts is among the most difficult NER tasks in *BioNLP*, investigated in a myriad of studies such as [14–16]. *EMU* is another mutation tagger effective in reducing the annotation time of articles candidate for mutation related associations [17]. It should be noted that implementing a state-of-the-art automated SNP and phenotype NER is not the objective of this research. Rather, it is the first step toward producing an association extraction corpus, where, the product of the automated algorithm is subsequently checked manually.

The rest of the paper is organized as follows: The next section reviews some of the related works; section three presents the methodology of the paper; section four is dedicated to the evaluation and results; and the last section concludes the paper.

### Related works

In this section, we are going to introduce some of the relevant works about preparing the datasets used for extracting mutation related entities including disease as well as different methods of annotating negation and levels of confidence in the biomedical domain.

### Mutation association extraction methods and corpora

Besides classical relation extraction tasks in the BioNLP domain such as protein-protein and gen-disease, certain novel methods and corpora have been developed with the aim of extracting mutation/polymorphism and disease associations, among which, mention can be made of *BRONCO* [18] and *Variome* [19]. BRONCO contains more than four hundred variants and their associations with genes, diseases, drugs and cell lines in the context of cancer, all extracted from 108 full-text articles. Variome covers 12 types of relations annotated in 10 full-text articles. While BRONCO includes more documents, both corpora annotate several types of relations, such as mutation-disease association, as binary relations on a full-text level. On the other hand, the advantages of abstract-level relation extraction over full-text were mentioned in the introduction section. Therefore, the prepared corpus in this research was provided on an abstract level.

*PKDE4J* [5] and *Dimex* [6] are two methods for extracting mutation and disease association, the latter being a rule-based unsupervised mutation-disease association extraction working on the abstract level. The PKDE4J, however, is a supervised method that employs a rich set of rules to detect the used features. Both methods work on usual binary relations that determine whether or not there exist an association; neither method considers the degree of certainty or confidence [20]. developed another related miner system that gathers heterogeneous data from a variety of literature sources in order to draw new inferences as to the target protein families. Likewise, Ravikumar and his colleagues [21] developed an automated extraction tool in order to obtain protein-specific residue associations from the literature. Another similar automated approach was proposed by [22], which extracts impacts and related information from literature. In another recent study, Klein et al. proposed the principal infrastructure for the benchmarking of mutation text mining systems [23].

The corpus prepared in this research was annotated with negation cues and scopes, modality markers, and neutral association candidates. Such linguistic features were conducive to the extraction of more accurate information about the extracted SNP-phenotype associations.

### Annotating the modality and degree of confidence

As mentioned earlier, “modality” indicates the degree to which a certain observation is possible, probable, likely, certain, permitted, or prohibited. A host of studies have been conducted for the identification of modality and speculation in NLP; very few, however, have been employed for the classification of modality language in bioscience texts.

Although several studies such as [24] have been conducted within the linguistics community as to hedging in scientific texts, in neither is there direct relevance to the task of classifying from an NLP and machine learning perspective.

Light and his colleagues conducted one of the very few direct studies [25], where the speculation identification is introduced using examples from the biomedical domain. They address the question of whether there is sufficient agreement among researches as to what constitutes a speculative assertion that renders the task viable from a computational perspective. Despite the fact that Light attempts to separate the two sides of speculation (strong and weak), he fails to glean sufficient evidence for such a reliable distinction. They conclude that having a reliable distinction between speculative and non-speculative sentences is feasible, and reliable automated methods might also be developed.

It is noteworthy that in addition to the preponderance of biomedical relation extraction annotations that merely include usual binary association information, there exist certain others containing extra-linguistic information including POS, negation, and speculations information. As an example, the Genia corpus [26], along with biological events, contains annotations for three levels of uncertainty. Nonetheless, to the best of our knowledge, all of the mutation related corpora have only been annotated with binary associations. In the current study, the corpus was enriched through adding more linguistic information such as the linguistic based confidence level of associations, modality markers, and neutral association candidates.

### Negation annotation

In general, two negation detection methods have been developed to annotate the employed corpora: A linguistic-based approach and an event-oriented approach. Among other negation annotated corpora, one may refer to the two most well-known: the linguistically-focused, scope-based BioScope [27] and the event-oriented *Genia* [26]. In *BioScope*, scopes recognize the position of the key negated event within the sentence, with each argument of the key events coming under the scope, as well. Genia, on the contrary, independently deals with modality within the events. In a Genia event, biological concepts (relations and events) are annotated for negation, yet no linguistic cues are annotated. In fact, the objective of the BioScope corpus is to approach this language phenomenon in a general, task-independent, and linguistically-oriented manner. It can further automatically recognize negation scopes and cues in sentences.

*NegDDI-DrugBank* is another corpus that was annotated by the authors of the previous work with scopes of negation and negation cues [28]. The automatic extraction of Drug-Drug interactions from the text is held to be highly significant, as two corpus versions (in 2011 and 2013) were prepared in this regard. Concerning the high rate of negated sentences in the DDI corpus, a complete set of sentences within DDI 2011 (with a total of 5806 sentences and 579 files) was automatically annotated with negation scopes and cues. The results were, then, manually checked by three experts to address possible mistakes within the course of the automated process [29]. Adding a new XML negation-tag containing negation cues and negation scopes, the NegDDI-DrugBank corpus was established.

### Corpus construction

**Fig. 3:**
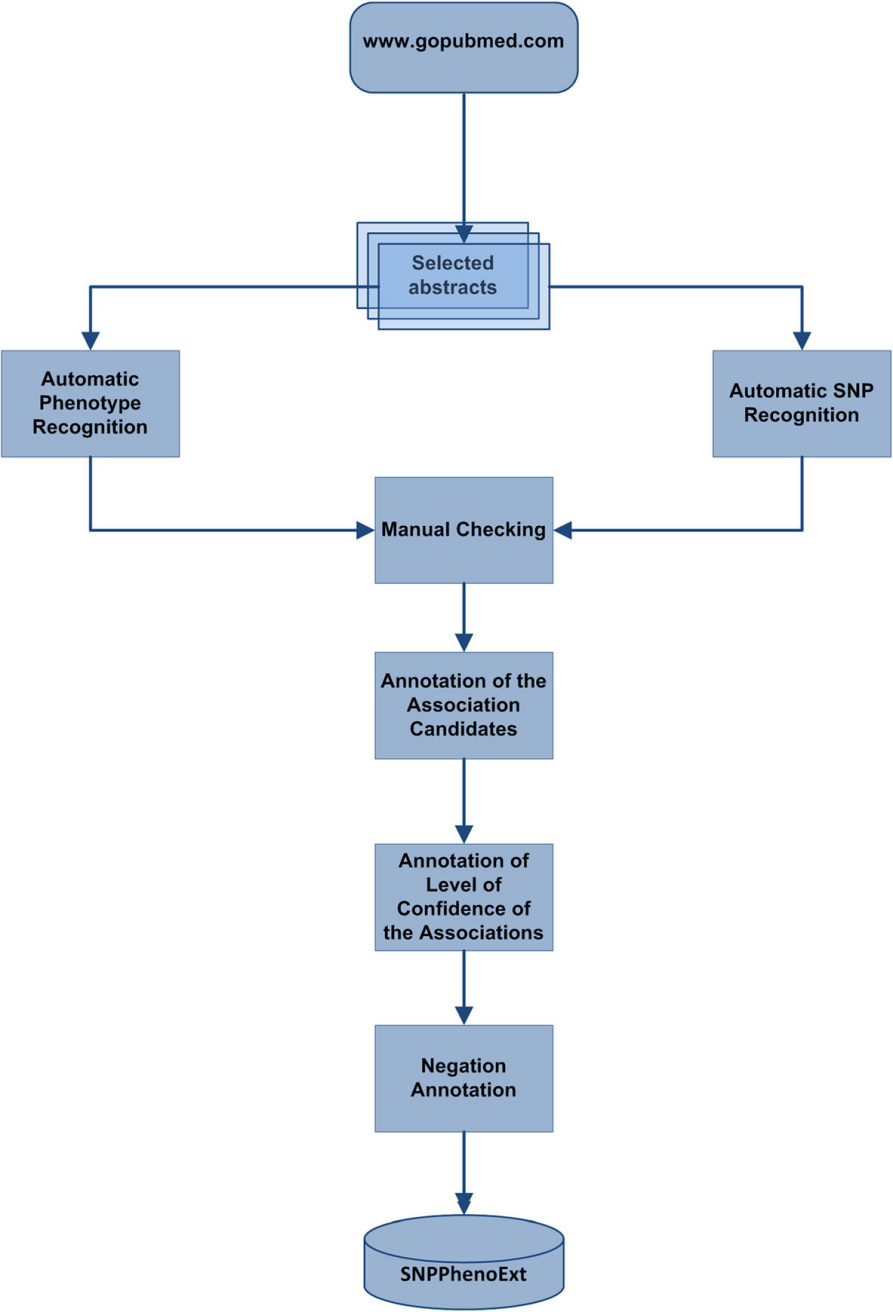
Different steps for producing the SNPPhenA corpus

In this section, the steps followed in the construction of the SNPPhenA corpus are explained. The entire process consists of three major steps of collecting documents, automatically and manually recognizing the SNP and phenotypes, and annotating the associations and the related information (Fig. 3). The last step entails annotating the association candidates, the confidence level of associations, the modality markers and the negation scopes and cues of the sentences.

In order to have consistent annotations, all annotators were given the same instruction which includes a pellucid definition of the entities and their relationships, rules and conventions of annotating the confidence level of associations and complete examples for each type of tags. The annotation guideline also contains rules for tackling linguistic phenomena such as negation cues and modality markers. Moreover, this document presents different types questions raised and retorted by the annotators during the annotation process. The annotation guideline can be found on the website of the corpus.

In the end, 360 XML files were generated comprised of the abstract texts, SNPs, Phenotypes, and the SNP-phenotype associations in the selected sentences. The Phenotypes, SNP names and the association candidates were annotated as xml element tags for each nominated sentence in the abstract. Next, the annotations and the final product were manually checked. The produced SNPPhenA corpus is available for public use 1. So as to better fathom and employ the corpus, brat stand-off annotation format of the files is also available at the website of the corpus. The next subsection is dedicated to the abstracts collection process 2.

### Abstract retrieval

Information provided by the “http://www.gopubmed.org/” search engine was used to collect genome-wide association abstracts. *GoPubMed* is a webserver allowing users to explore PubMed search results with Gene Ontology [30]. Twenty popular SNPs were used as query terms enumerated popular by “http://www.snpedia.com/”website; the extracted list of abstracts was shortened via selecting those comprised of popular disease names. The list was finally truncated again through choosing those that have candidate sentences consisting of both types of entities. We collected a total of 360 abstracts (including 2625 sentences) with at least one candidate sentence with an SNP and a phenotype name. There were 483 key sentences containing at least one SNP and one phenotype name that were annotated with the xml element “SENTENCE”. The total number of SNP names annotated in the SNPPhenA corpus was 875. It is worth mentioning the SNPPhenA is a sentence-level corpus and sentences merely including SNP or Phenotype were not annotated.

The next step was to perform an automatic Named Entity Recognition, followed by a manual checking of sentences with candidate relations for SNPs and phenotype names, as explained in the section below.

### Named entity recognition (NER)

**Fig. 4:**
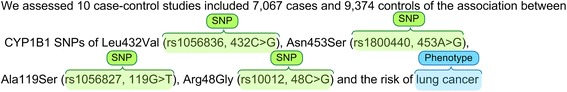
A sample of SNP and phenotype named entity recognition in the corpus

An essential part of biomedical NLP is to detect biomedical named entities [31]. During the construction process, two Named Entity Recognitions were done on SNPs and Phenotypes. These two tasks are minutely explained in the two following subsections. A sample of implemented NERs is shown in Fig. 4.

### Phenotype NER

A phenotype is the appearance of an organism in terms of its morphology, development, physiology, behavior and its concomitant products [3]. Although there are databases containing disease names and popular phenotype names, no compendious database of phenotypes is yet available.

In this regard, a dictionary-based NER task was implemented by combing two more complete and pertinent databases. The prepared dictionary includes a list from the Comparative Toxicogenomics Database (CTD) for disease names [32]. Also included is the phenotype ontology prepared in the blast project [33]. The collected list of phenotypes includes 65,530 phenotype names along with more than twelve thousand disease names and their synonyms.

The phenotype names were initially recognized automatically by the prepared dataset. Manual checks were subsequently made by two experts in order to identify missed or inexact phenotypes.

A short list of the most frequent phenotypes is shown in Table 1 where the top two phenotypes in the corpus are “health risk” and “smoking”.

**Table 1:** Some of the most occurred phenotypes in the corpus

Phenotype/phenotypic trait	Num. of abstracts
health risk	40
smoking	33
Obesity	25
metabolic syndrome	16
hypertension	10
insulin sensitivity	9
hypertriglyceridemia	7
glucose metabolism	6
impaired glucose tolerance	5
longevity	4
body mass intake	4
cognitive performance	4
skin pigmentation	3
AIDS	3

### SNP NER

The inconsistent description of biological data elements renders the relation extraction tasks challenging. Names associated with polymorphism are particularly problematic because historical or common names are, more often than not, employed instead of standard nomenclature [34], specifically in candidate gene association studies. What is more, it is hard to find the links between historical or common SNP names and refSNP [35]. To address this issue, we implemented a database containing both refSNP(rs) and historical names, matched with their corresponding *rsID* numbers, while utilizing the *Variant Name Mapper*(VNM) tool [36]. The VNM tool consists of historical names matched with their corresponding rsID numbers extracted from multiple open-access databases, including SNP500Cancer [37], SNPedia [38], pharmGKB [39]. The database was utilized for extracting the different SNP names.

Similar to the phenotype NER process, SNP name annotations were initially checked manually by two biology experts and verified by a third professional annotator. A short list of the most frequent SNPs is shown in Table 2.

**Table 2:** Eight of most occurred SNP’s in the SNPPhenA corpus and number of contained abstracts

SNP	Number of abstracts
rs12255372	78
rs429358	55
rs7412	46
rs4680	38
rs1051730	25
rs662799	20
rs1799971	18
rs1800629	14

### Annotating the candidate SNP-phenotype associations

This section deals with the process of annotating the associated candidates which includes the annotation of the SNP-phenotype associations, the confidence level of associated candidates, modality markers, and negation scopes and cues in the negated sentences.

### Annotating the SNP-phenotype associations

**Fig. 5:**
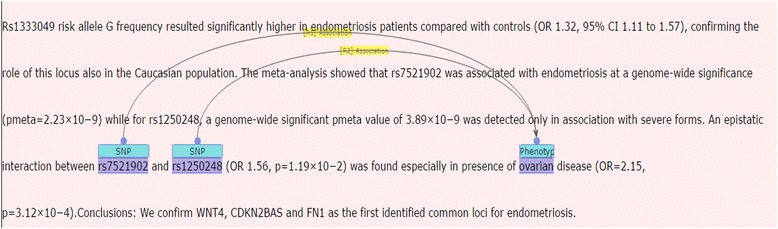
A sample of two annotated associations between two SNPs and a phenotype in the SNPPhenA corpus

**Fig. 6:**
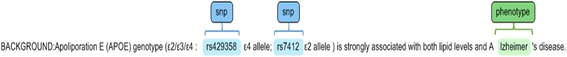
Samples of positive association candidate between highlighted two SNPs and a phenotype

**Fig. 7:**
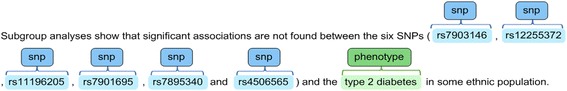
Samples of negative association candidate between highlighted six SNPs and a phenotype

**Fig. 8:**
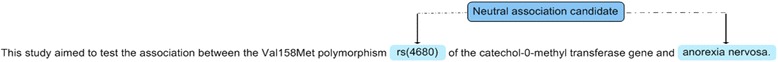
A sample of neutral association candidate with used highlighted entities

Following the collection of abstracts and the determination of the SNP and phenotype candidate names, the associations between SNP and phenotype were manually annotated by three gurus in genetics (Fig. 5). The SNP-phenotype candidates were classified into three categories of positive, negative and neutral. The positive SNP-phenotype relation candidates are those with clearly indicated associations (Fig. 6). In contrast, negative SNP-phenotype relation candidates are those in which a lack of association is evident (Fig.7). In addition to the typical classes of relationships, a neutral class is defined for those that fall between the two other classes, where the presence or absence of association is not remarked in the sentence (see Fig. 8).

As Fig. 8 shows, the presence or absence of association is neither mentioned between “rs4689” and “anorexia nervosa”, nor can it be identified with a high level of confidence, hence, the association between the SNP and the phenotype was annotated as neutral.

In more precise terms, an SNP-Phenotype association candidate is identified as neutral if:

(i) The absence or presence of association between SNP-phenotype cannot be specified from the sentence (or container clause) with a confidence level of more than zero.

(ii) The status of presence or lack of association between the SNP and the phenotype does not change from positive to negative or vice versa if the sentence (or container clause) is negated and SNP and phenotype names are located in the scope of the negation.

(iii) The confidence level of association between SNP and the phenotype does not change if a modal marker is utilized in the sentence and both entities are located in the scope of modality.

**Fig. 9:**
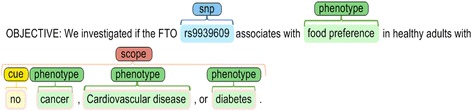
A sample of neutral association candidate with a negation cue

The association in Fig. 9, for instance, is neutral and the used negation cue (“no”) does not change the status of the association between the SNP and the phenotypes.

It is worth mentioning that in most relation extraction corpora, neutral candidates were considered to be part of the negative (non-positive) class. Considering them as a separate class of associations allows researchers to conduct different types of experiments. More details as to the role of neutral candidates in biomedical relation extraction tasks can be found in the author’s other study [40].

Similar to the previous steps, the manual checking was initially performed by two experts, and in order to sort out the issue of contradictory confidence levels, the verdict of a third expert annotator was taken into account.

### Annotating the level of confidence of the SNP-Phenotype associations

In spite of the fact that genetic components have the instructions for the growth and development of each individual, a person’s phenotype is influenced by environmental factors during embryonic development and throughout life. Environmental factors can stem from a variety of influences such as diet, climate, illness and level of stress. For instance, the capability to taste food is a phenotype estimated, by scientists, to be 85% influenced by genetic inheritance [41]. Nevertheless, environmental factors such as dry mouth or recently eaten food could affect such ability.

“Phenotypic plasticity” is the ability of a genotype to generate more than one phenotype due to various environments [42]. The plasticity is considered to be high if environmental factors have a strong influence. Conversely, if the phenotypic plasticity is low, the genotype can be made use of so as to reliably predict the phenotype. The degree of influence environmental factors have on a person’s ultimate phenotype is, not infrequently, a matter of heated scientific debate.

Differing phenotypic plasticities alongside possible unknown genetic components are the two reasons why GWA study uses confidence level in order to describe the strength of association. The linguistic-based confidence level of the reported association ultimately yields informative data leading to the determination of phenotypic plasticity.

However, there is no available data source or automated method for extracting confidence level from the obtained results. This is when the presence of such a tool and data source is critical and conducive to reviewing literatures.

For this purpose, the confidence levels of positive association candidates in the corpus were annotated by a guru in human genetics. Based on the strength of the linguistic correlation between each individual phenotype and the relevant SNP mentioned in the abstract, the confidence level of associations was categorized into weak, moderate, and strong. Moreover, when the association is neutral (ASSOCIATION = neutral), the degree of confidence is set to “zero”. The confidence levels were assorted considering modality, adverbs and the reported statistical results (p-value). Detailed information about the annotation guidelines can be seen in the guidelines document, available on the website of the corpus. The process, all the same, is demonstrated here via some samples.

**Fig. 10:**
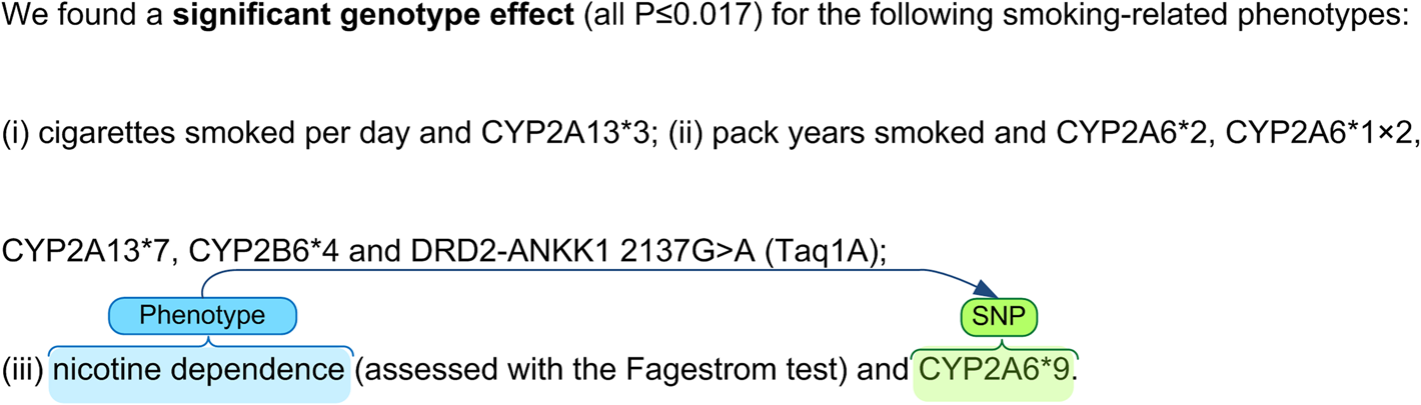
A sample of a strong association that has been mentioned to have a strong degree of confidence

The sentence shown in Fig. 10, for example, is considered to have a high confidence level as it indicates “found a significant genotype effect”.

**Fig. 11:**
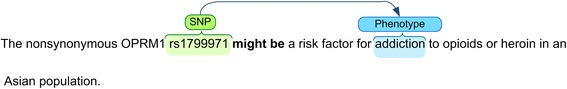
A sample of a weak association that has been mentioned to have a weak degree of confidence

**Fig. 12:**
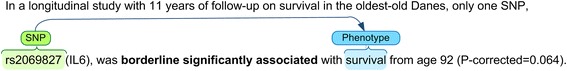
A sample of moderate association that has been mentioned to have a moderate degree of confidence

The sample mentioned in Fig. 11, on the other hand, is annotated as having a weak confidence level because of the “might be” clause. However, there exist certain cases that fall under both two categories such as the sample below (see Fig. 12), annotated as moderate.

The annotation of confidence level was carried out by two biology experts both of whom had the same opinion regarding 86% of the association candidates in the whole corpus. In order to sort out the issue of contradictory confidence levels (14%), the opinion of a third guru annotator was considered.

### Linguistic based negation detection and modality markers

**Fig. 13:**
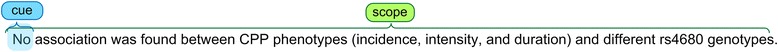
A sample of a negated sentence with negation cue and scope

Identifying negative statements is essential in order to obtain accurate information from the text data. The sentence in Fig. 13 demonstrates the importance of considering negation where there is no association between “APOE (rs429358)” and “bvFTD”; however, if the negation had been neglected, an incorrect association might have been identified.

A rule-based system, proposed by [43], was initially utilized in order to annotate the negation scopes and cues. During the process, a set of negation cues such as “not”, “lack”, were detected making use of Bioscope’s guidelines. Negation cues indicate that a negation exists in a sentence. Considering the syntactic context, the scopes of negation and negation cues were subsequently determined, a task already done in a previous work by the authors [28] annotating the DrugDDI 2011 corpus. In order to preclude any possible mistakes, manual checks were made by an expert following the automated process.

In addition to the negation cue and scopes, modality markers were annotated during the annotating process. The employed modality markers obtained from the list were already provided in [44], which is an extension of the list provided by [45] for the biomedical domain. The process includes an automated annotation, followed by an expert performing the manual check. The five more frequent annotated modal markers in the corpus are: “suggest”, “more”, “strong”, “observe”, and “show”.

## Evaluation and results

In this section, inter-annotator agreement analyses and the calculated scores are initially presented; then some of the basic statistics of the produced corpus will be demonstrated; and finally, the results obtained from our first experiment using the corpus are presented.

### Inter-annotator agreement

In order to evaluate the quality of the corpus and the reliability of the annotations, the inter-annotator agreement score was measured for the task of classifying candidate sentences into positive, negative and neutral classes, and also for the task of determining the confidence level of the association. As was mentioned before, two annotators had independently tagged the corpus. In the case of disagreement between two tags, a third annotator was asked to decide on the correct one. For the task of classifying candidate sentences, inter-annotator agreement was 91%, which means that in 91% of cases, the two annotators agreed. Additionally, we computed Cohen’s Kappa coefficient [46] for the two annotators; this coefficient takes into account the degree of agreement that could be expected to occur by chance and is computed as follows:

$$ \kappa = \frac{p_o-{p}_e}{1-{p}_e} $$

Where P_o_ is the relative observed agreement among annotators, and p_e_ is the hypothetical probability of chance agreement. The Kappa value was 0.79 for the two annotators. In general, κ = 1 indicates a complete agreement. Furthermore, κ < 0 shows that there is no agreement between annotators other than what would be expected by chance (as given by p_e_).

As far as the task of annotating the confidence level of the association with four categories (zero, weak, medium, strong), annotators agreed in 87% of the occasions; yet the Kappa value was 0.80 which is satisfactory.

### Characteristics of the SNPPhenA corpus

This section provides detailed statistics as to the linguistic and nonlinguistic properties of the corpus. The basic properties of the corpus are presented in Table 3 which includes the statistics of the produced corpus in terms of test and training parts. As the table shows, the candidates with a positive association comprise the largest category while the negatively associated candidates constitute the smallest category.

**Table 3:** Basic statistics of the SNPPhenA corpus in terms of test and train parts

Item	Train	Test	Total
Files	270	90	360
Sentences	1940	685	2625
Key sentences	362	121	483
SNP	691	244	935
Phenotypes	496	158	654
SNP-Phenotype association candidates	935	365	1300
Neutral candidates	142	166	308
Negative candidates	91	29	120
Positive candidates	702	170	872

Table 4 provides the detailed analyses concerning the different types of SNP-phenotype association candidates.

**Table 4:** Statistics of different types of SNP-phenotype association candidates in the SNPPhenA corpus

Item	Number	Percentage (%)
Total SNP-phenotype association candidates	1300	100
Candidate with at least one negation cue	218	16.8
Candidates with only one negation cue	188	14.5
Candidates with clause connectors	823	63.8
Candidates without clause connector	470	36.2
Weak degree of confidence candidates	515	39.6
Moderate degree of confidence candidates	124	9.5
Strong degree of confidence positive candidates	233	17.9

Additionally, as mentioned earlier, the key negated sentences in the corpus were annotated with scopes of negation and negation cues. As Table 4 shows, 16.8% of the sentences have at least one negation cue. Further analysis shows that “not” and “no” with respective occurrences of 35 and 38 were the most frequent negation cues. According to the conducted analyses, each sentence in the corpus had an average of 76.9 tokens, 1.7 SNPs, and 1.2 phenotypes.

As illustrated in Table 3, 76.3% of the samples are distinguished (i.e. they are positive and negative association candidates). It can, therefore, be concluded that the annotated sentences were mostly expressed as a direct mechanism or association between one or more SNPs and a phenotype.

Additionally, as Table 4 shows, 63.8% of the candidate sentences have at least one clause connector, while 36.2% do not have one. The result of statistical analysis on the clause connectors further indicates that 9.7% (=87/895) of instances had concessive clauses.

### Experiment

The results of our first experiments with the corpus are presented in this subsection. Although several mutation-related association extraction methods have recently been developed, automatically measuring the confidence level in an association is a novel task. Consequently, our first experiments were evaluated via certain baseline kernel methods for the two subtasks.

In order to categorize the associations, we employed the two kernel methods that have been expansively made use of in the relation extraction task; the local context kernel [47] and sub-tree kernel [48]. Additionally, the binary Bag of Word (BOW) method was carried out on the corpus so as to predict the degree of confidence for the associations. In all the experiments, the training part of the prepared corpus was used for training the classifier and the test part was employed for testing the system (Tables 5, 6 and 7).

**Table 5:** Comparative f-score results for the test SNPPhenA part for two kernel methods with all types of candidates (positive, negative and neutral class)

Method	LCK	Subtree kernel
F1	71.3%	57.7%
Recall	68.7%	51.8%
Precision	69.2%	50.3%

**Table 6:** Obtained comparative results for the test SNPPhenA corpus for the two investigated kernel methods with non-neutral candidates (positive and negative class)

Method	LCK	Subtree kernel
F1	63.4%	45.7%
Recall	59.8%	41.3%
Precision	56.6%	40.1%

**Table 7:** Obtained results for the calculating confident interval of the positive association of the test part of the SNPPhenA corpus by bag of words method

Parameter	Weak degree of confidence	Moderate degree of confidence	Strong degree of confidence
F1	69.5%	32.6%	35.3%
Recall	66.4%	30.5%	34.2%
Precision	65.3%	31.6%	32.2%

Table 5 shows the performance of the two utilized baseline methods, applied to all three types of candidates. The reported f-score was measured for the detection of positive SNP-phenotype association candidates. Table 6 further indicates the performance of the baseline methods were only applied to the positive and negative association candidates.

The results of the confidence level prediction of associations are presented in Table 7 where the best f-measure is related to the candidate expressions of associations with a weak confidence level, while the worst result is obtained for the moderate confidence level.

The lower performance of identifying the confidence level of association in comparison with the association extraction method demonstrates that the simple features used in the binary BOW may not have enough information to surmount the task and more linguistic features are required. Moreover, the difficulty of the task might be precipitated by the fact that during the annotation process, the annotators employed the mentioned p-value number as a complementary factor for identifying the confidence category, which was the case with 20% of the candidate sentences. It can, accordingly, be concluded that accurately identifying ranked association from biomedical articles requires more linguistic features including dependency parsing, lemmatizing and features related to identifying the significance degree of the biomedical statistical tests.

A simple version of the baseline method can be found online 3. It is indispensible to mention that the online system may have a worse performance in comparison with the reported results in this section due to the absence of manual checking during the NER task as well as the omission of the negation detection step.

All the kernel method experiments were carried out by a support vector machine with SMO [49] implementation. Weka API [50] was used as the implementation platform.

## Conclusion and future work

In this research, a SNPPhenA corpus was developed in order to extract the ranked associations of SNPs and phenotypes from GWA studies. The process entailed collecting relevant abstracts, Named Entity Recognition, and annotating the associations, negation, modality markers, and the confidence level of the associations.

As opposed to the previous biomedical relation extraction corpora containing true and false types of relations, the annotated associations in the corpus were divided into three classes: positive, negative and neutral candidates. The neutral candidates were those SNP-phenotype candidates that showed no clear evidence as to the presence or lack of association between the SNPs and phenotypes. Identifying neutral candidates is critical for the negation process as the status of such candidates and their corresponding level of confidence do not change when they are located in the scope of negation terms; the status of distinguished association candidates, on the other hand, change in such cases. Similarly, the confidence level, certainty or uncertainty of a neutral candidate, does not change if it is located in the scope of a speculation or modality term. Hence, determining the effect of negation as well as modality terms requires the identification of neutral candidates.

Not to be forgotten is the fact that the SNPPhenA corpus must be considered as an initial step in extracting graded associations from literature, which could result in the idea of a fuzzy relation extraction task that can be employed so as to construct better biomedical ontologies.

Furthermore, it is important for future researches to employ more linguistic-based and non-linguistic-based factors that could be utilized to determine the confidence of the reported associations. Credibility of the genotyping techniques (such as MLPA or RFLP) and the validity of the research through graph-based network analyses can be employed in the process of identifying the overall confidence level of the reported associations.

## Additional file

Additional file 1: Abstract files of SNPPhenA corpus. (ZIP 651 kb)

## Footnotes

1
https://figshare.com/s/b18f7ff4ed8812e265e8

2
https://figshare.com/s/f19191317056d6835b38

3
http://snpphenotypeext-nilg.rhcloud.com/
