# Supplementary material for: SNPPhenA: a corpus for extracting ranked associations of single-nucleotide polymorphisms and phenotypes from literature
Source: J Biomed Semantics. 2017 Apr 7;8:14. doi: 10.1186/s13326-017-0116-2 (PMC5383945; doi:10.1186/s13326-017-0116-2)
Supplement: Additional file 1: — Abstract files of SNPPhenA corpus. (ZIP 651 kb) [file 13326_2017_116_MOESM1_ESM.zip › Certification letter English Edit 2.pdf]

CERTIFICATE

Of English Editing

CERTIFIED

This is to certify that the article titled

SNPPhenA: A corpus for extracting ranked associations of single-nucleotide polymorphisms and phenotypes from literature

has been edited for language by the native English editorial board of Research Editor

ایده پردازان میلاد پارس
ثبت : ۳۶۲۵۷

Research Editor CEO
11/23/2016

Montakhab Building, Zand BLVD,Shiraz, Iran
Postal Code:7135654369
Tel:+98-713-2352763
http://ResearchEditor.ir

Research EDITOR
